# Activation of Bisulfite with Pyrophosphate-Complexed Mn(III) for Fast Oxidation of Organic Pollutants

**DOI:** 10.3390/ijerph19159437

**Published:** 2022-08-01

**Authors:** Qianli Guo, Xianhu Qi, Jian Zhang, Bo Sun

**Affiliations:** 1 Shandong Key Laboratory of Water Pollution Control and Resource Reuse, School of Environmental Science & Engineering, Shandong University, Qingdao 266237, China; guoqianli2002@163.com (Q.G.); qixianhu735@163.com (X.Q.); zhangjian00@sdu.edu.cn (J.Z.); 2 College of Safety and Environmental Engineering, Shandong University of Science and Technology, Qingdao 266590, China

**Keywords:** Mn(III), bisulfite, advanced oxidation processes, sulfate radical, micropollutant abatement

## Abstract

Aqueous complexes of Mn(III) ion with ligands exist in various aquatic systems and many stages of water treatment works, while HSO_3_^−^ is a common reductant in water treatment. This study discloses that their encounter results in a process that oxidizes organic contaminants rapidly. Pyrophosphate (PP, a nonredox active ligand) was used to prepare the Mn(III) solution. An approximate 71% removal of carbamazepine (CBZ) was achieved by the Mn(III)/HSO_3_^−^ process at pH 7.0 within 20 s, while negligible CBZ was degraded by Mn(III) or HSO_3_^−^ alone. The reactive species responsible for pollutant abatement in the Mn(III)/HSO_3_^−^ process were SO_4_^•−^ and HO^•^. The treatment efficiency of the Mn(III)/HSO_3_^−^ process is highly related to the dosage of HSO_3_^−^ because HSO_3_^−^ acted as both the radical scavenger and precursor. The reaction of Mn(III) with HSO_3_^−^ follows second-order reaction kinetics and the second-order rate constants ranged from 7.5 × 10^3^ to 17 M^−1^ s^−1^ under the reaction conditions of this study, suggesting that the Mn(III)/HSO_3_^−^ process is an effective process for producing SO_4_^•^^−^. The pH and PP:Mn(III) ratio affect the reactivity of Mn(III) towards HSO_3_^−^. The water background constituents, such as Cl^−^ and dissolved organic matter, induce considerable loss of the treatment efficiency in different ways.

## 1. Introduction

Under the stress of population expansion and increasing urbanization, water scarcity strongly increases [1,2]. The situation is becoming worse due to the water quality deterioration. To address this issue, various strategies were proposed, such as recycling of waste to reduce the pollution of environment [3,4,5]. However, many micropollutants are not disposed reasonably and are inevitably discharged into water. These micropollutants, including pharmaceuticals and personal care products (PPCPs), pesticides, biocides, and many others, lead to human health concern [6,7]. Degrading/removing micropollutants has become one of the most important tasks in supplying safe drinking water. However, some of them resist conventional treatment processes, including coagulation, sedimentation, filtration, chlorination, and biological treatment [8]. For these recalcitrant micropollutants, their oxidative degradation is often achieved by the advanced oxidation processes, which produce highly reactive species such as HO^•^ and SO_4_^•−^ through activating the radical precursor with different strategies [9,10,11,12].

Over the past decades, SO_4_^•−^-based AOPs have drawn significant attention as a viable alternative to traditional HO^•^-based AOPs in water and wastewater treatment [10] due to their various virtues, including (i) the higher redox potential ${(E}_{{\mathrm{SO}}_{4}^{•-}{/\mathrm{SO}}_{4}^{2-}}^{0}=+2.60–+3{.10\;V}_{\mathrm{NHE}}$ > $E_{{\mathrm{HO}}^{•}{/\mathrm{HO}}^{-}}^{0}=+1.90–+2{.70\;V}_{\mathrm{NHE}})$ [13], (ii) lower costs of storage and transportation of persulfate, the common SO_4_^•−^ precursor, than H_2_O_2_, the common HO^•^ precursor, (iii) the higher achievable radicals formation yield of persulfate [14,15,16,17], and (iv) a wider variety of methods available to activate persulfate. The commonly used persulfate contains peroxydisulfate and peroxymonosulfate, both of which could be activated by photo radiation, heat, base, organic compounds, transition metals, and some composites (e.g., Co_3_O_4_/C_3_N_4_) [13,18,19,20,21,22,23,24,25]. However, the application of peroxydisulfate and peroxymonosulfate in water treatment might be limited due to their expensive reagent price and residual peroxide ions. Therefore, new attempts were made to seek greener and more cost-effective precursors of SO_4_^•−^.

Recently, activation of S(IV) (i.e., HSO_3_^−^ or SO_3_^2−^) to produce SO_4_^•−^ using transition metal or metal oxides (e.g., Fe(III), Fe(IV), MnO_2_, MnO_4_^−^, Cr_2_O_7_^2−^) has been developed to remove micropollutants (Equations (1)–(3)) [26,27,28,29,30,31,32,33,34]. Due to their low cost, nontoxicity, convenient operation, and high efficiency, S(IV)-based AOPs are supposed to be excellent methods for producing SO_4_^•−^. Moreover, the residual S(IV) in water can be removed by aeration, leading to the formation of nontoxic sulfate.

Transition metal + HSO_3_^−^/SO_3_^2−^ → SO_3_^•−^ (1)

SO_3_^•−^ + O_2_ → SO_5_^•−^ (2)

SO_5_^•−^ + HSO_3_^−^/SO_3_^2−^ → SO_4_^•−^ + SO_4_^2^^−^ (+ H^+^) (3)

Among the proposed transition metal or metal oxides, Mn(IV) is the thermodynamically favored oxidation state in surface waters and is nearly ubiquitous, and thus gains considerable attention [35]. Mn(IV) was commonly considered to exist in the form of MnO_2_. However, it was found that treatment efficiency of the MnO_2_/HSO_3_^−^ process is highly dependent on the morphology of the employed MnO_2_. Sun et al. [29] reported that the colloidal MnO_2_ activated HSO_3_^−^ process led to the removal of pollutants at the timescale of tens of seconds. Differently, Wang et al. [31] found that amorphous MnO_2_ activated HSO_3_^−^ process resulted in the half-time of pollutant degradation at several hours. It was demonstrated that the reactivity of MnO_2_ towards contaminants is highly related to the content of Mn(III), and Mn(III) was considered to possess higher redox activity than MnO_2_ [36,37,38]. However, the influence of Mn(III) on activating HSO_3_^−^ is unknown. In addition to being absorbed by MnO_2_ particles, Mn(III) stabilized by ligands (free Mn(III) ion is prone to disproportionation) is widespread in natural waters and water treatment processes. Trouwborst et al. [39] found that soluble Mn(III) could be stabilized by natural ligands and its concentration was as high as 5 µM, which constituted up to 100% of the total dissolved Mn pool in the Black Sea. Madison et al. [40] disclosed that soluble Mn(III) is primarily produced via oxidation of Mn(II) diffusing upwards from anoxic sediments in the Laurentian Trough (Quebec, Canada). Mn(III) was also reported to be produced through the interaction of influent Mn oxide solids with natural organic matter (NOM) in the clarifier sludge of a water treatment plant in England [41]. Thus, the added HSO_3_^−^ seems very likely to encounter with Mn(III), and single electron abstraction transfer from HSO_3_^−^ to Mn(III) was expected, leading to the following formation of SO_4_^•−^ (Equations (1)–(3)).

However, the performance of the Mn(III)/HSO_3_^−^ process for pollutant abatement was unclear, impeding not only the understanding of the already-constructed MnO_2_/HSO_3_^−^ process but also the design and development of a new Mn(III)/HSO_3_^−^ advanced oxidation process. To address this, efforts were needed to evaluate the potential of the Mn(III)/HSO_3_^−^ process in pollutant abatement, which is the aim of this study. Firstly, the efficiency of pollutant removal by this process as the function of reaction conditions (e.g., pH, dosage of Mn(III), HSO_3_^−^, dissolved oxygen, and ligand:Mn(III) ratio) was investigated. Then, the kinetics of Mn(III) reacting with HSO_3_^−^ were analyzed. Further, the reactive species responsible for pollutant degradation in the Mn(III)/HSO_3_^−^ process were differentiated. Finally, the influence of common background water constituents (e.g., Cl^−^ and NOM) on pollutant degradation by the Mn(III)/HSO_3_^−^ process was elaborated. Carbamazepine (CBZ) is one of the pharmaceuticals most frequently detected in the aqueous environment and was selected as the probe contaminant in this study [42].

## 2. Materials and Methods

### 2.1. Chemicals and Materials

Nitrobenzene (NB), pyrophosphate (PP), manganese sulfate (MnSO_4_), potassium permanganate (KMnO_4_), methanol (MeOH), and tert-butanol (TBA) were purchased from Sinopharm Chemical Reagent Co. Ltd., (Beijing, China). Sodium thiosulfate pentahydrate (Na_2_S_2_O_3_•5H_2_O), sodium bisulfate (NaHSO_3_, S(IV)), and sodium chloride (NaCl) were received from Macklin Biochemical Co., Ltd., Shanghai, China. Bisphenol A (BPA), CBZ, and 5,5-dithiobis(2-nitrobenzoic acid) (DTNB) were obtained from Aladdin Industrial Co., Ltd., Shanghai, China. All the above chemicals are of analytical grade and can be used directly without further purification. Humic acid (industry pure) was obtained from Sigma-Aldrich. The concentration of humic acid stock solution was determined with total carbon analyzer and used to simulate dissolved organic matter (DOM) in water. All of the experimental solutions in this study were prepared by dissolving chemicals in ultrapure water (18.2 MΩ-cm) produced using a ULUPURE water purification system, and stored at 4 °C in the dark. The stock solutions of NaHSO_3_ (250 mM) were freshly prepared every day to avoid oxidation by oxygen. The species of S(IV) depend on pH (${\mathrm{pK}}_{{a,\mathrm{HSO}}_{3}^{–}}$= 7.2), and HSO_3_^−^ was used to represent S(IV) in this study.

Free Mn(III) ion is prone to disproportionation to yield MnO_2_ and Mn(II) under environmentally relevant conditions. Most of the past studies about Mn(III) were conducted with insoluble Mn(III)-rich Mn oxides or in the presence of excess ligands. In this study, PP, a nonredox active ligand, was employed to stabilize Mn(III), and the stability constants were reported to be at least 10^6^. The stable Mn(III) stock solution was prepared with the following procedure: a mixture of 10 mL of KMnO_4_ (50 mM), 1.25 mL of Na_2_S_2_O_3_ (0.2 M), and 100 mL of PP (0.25 M), diluted to 1 L with ultrapure water with rapid mixing. Then, a pale red solution was obtained and remained stable for several days. The reaction that took place under these conditions can be described by Equation (4):

2MnO_4_^−^ + S_2_O_3_^2−^ + PP + 3H_2_O → Mn(III)-PP + SO_4_^2^^−^ + 6 OH^−^ (4)

### 2.2. Experimental Procedures

Batch experiments: The experiments on pollutant oxidation by the Mn(III)/HSO_3_^−^ process were conducted in 0.2 L glass bottles at 20 ± 1.0 °C. A piece of glass was used as the cover to prohibit the volatilization of organics. Reactions were initiated by quickly spiking Mn(III) into solutions containing target compounds, HSO_3_^−^, and the constituents(s) of interest. After reaction of 20 s, 1 mL of sample was rapidly transferred into a 2 mL sample vial containing 10 µL of Na_2_S_2_O_3_ stock solution. For the experiments where the effect of oxygen was to be examined, the reaction solution was sparged with N_2_ for at least 30 min before adding Mn(III) stock solution. All experiments were run in duplicates or triplicate, and the data were averaged.

Stopped-flow kinetic experiments: The reduction kinetics of Mn(III) by HSO_3_^−^ under different conditions were conducted with a stopped-flow spectrophotometer (SFS, Model SX 20, Applied Photophysics Ltd., Leatherhead, UK). Two working solutions were prepared before the experiments. One working solution contained 100 µM Mn(III) and 5 mM PP, and the other contained 500 µM HSO_3_^−^ and substrates of interest. Reactions were initiated by simultaneously injecting an equal volume of two working solutions. 258 nm was used to detect the decay of Mn(III) ($\varepsilon_{\mathrm{Mn}\left(\mathrm{III}\right)-\mathrm{PP}\;\mathrm{at}\;258\;\mathrm{nm}}{=6750\;M}^{-1}s^{-1}$) [28].

### 2.3. Analytical Methods

The concentrations of organic pollutants were analyzed by a high-performance liquid chromatograph (Thermo Scientific U3000 series) equipped with a UV detector and a C18 column (4.6 × 250 mm, 5 μm particle size, Thermo Scientific, Waltham, MA, USA) at 35 °C. The injection volume was 10 μL and the flow rate was 1.0 mL/min with a mobile phase of acetonitrile–formic acid aqueous solution at pH 3.5. The concentration of HSO_3_^−^ was monitored using the modified 5.5′-dithiobis(2-nitrobenzoic acid) colorimetric method [43]. The concentration of dissolved oxygen (DO) was measured online with a JPB-607A portable DO meter (Leici, Shanghai, China). Chlorine and chlorate were detected by ion chromatograph (DIONEX AQUION RFIC).

## 3. Results and Discussion

### 3.1. Pollutant Removal by the Mn(III)-Activated HSO_3_^−^ Process

**Figure 1:**
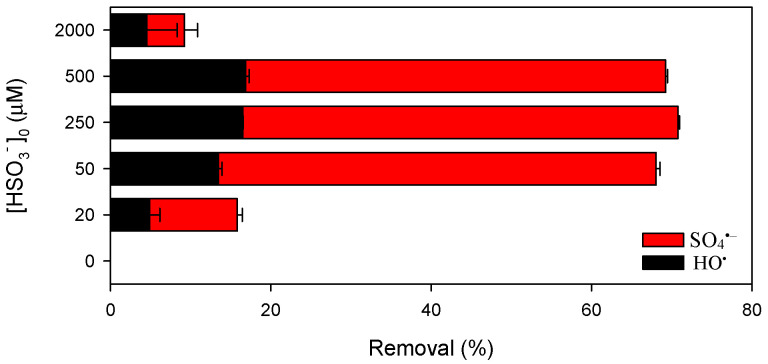
Influence of the dosage of HSO_3_^−^ on the removal of CBZ by Mn(III) at pH 7.0. Conditions: [CBZ]_0_ = 5 µM, [NB]_0_ = 1 µM, [Mn(III)]_0_ = 50 µM, [PP]_0_ = 2.5 mM, reaction time = 20 s.

Figure 1 shows the influence of HSO_3_^−^ on CBZ removal by Mn(III) at pH 7.0. Negligible CBZ was degraded by Mn(III) alone (Figure 1) and HSO_3_^−^ alone (not shown). Once HSO_3_^−^ coexisted with Mn(III), CBZ was degraded rapidly, suggesting the generation of reactive species in the Mn(III)/HSO_3_^−^ process. The removal of CBZ by the Mn(III)/HSO_3_^−^ process is highly related to the dosage of HSO_3_^−^. The removal of CBZ increased followed by decrease with increasing HSO_3_^−^ dosage from 20 to 2000 µM, and the maximal removal of 71% was obtained at HSO_3_^−^ dosage of 250 µM.

According to the valence states of manganese, the only product of Mn(III) reduction by HSO_3_^−^ was Mn(II), which is inert to CBZ. The reactive species in the Mn(III)/HSO_3_^−^ process are possibly derived from HSO_3_^−^ evolution. Based on the reported mechanisms of HSO_3_^−^ oxidation by dissolved oxygen in the presence of transition metals (Figure S1), various radicals (e.g., SO_3_^•−^, SO_4_^•−^, SO_5_^•−^, HO^•^) are expected to be involved in the Mn(III)/HSO_3_^−^ process. SO_3_^•−^ and SO_5_^•−^ are very weak, with redox potential of 0.63 and 1.1 V vs. NHE [44], and CBZ is recalcitrant to these two radicals [45]. The remaining candidates of reactive species that accounted for the CBZ oxidation by the Mn(III)/HSO_3_^−^ process are SO_4_^•−^ and HO^•^. Quenching experiments were conducted to verify the presence of these two radicals. MeOH and TBA were employed as the scavengers of radicals because TBA is only reactive to HO^•^ ($k_{{\mathrm{HO}}^{•}-\mathrm{TBA}}$ = 3.8 – 7.6 × 10^8^ M^−1^ s^−1^) [14], while MeOH is an effective quenching agent for both HO^•^ and SO_4_^•−^ ($k_{{\mathrm{HO}}^{•}-\mathrm{MeOH}}$) = 8.0 − 10 × 10^8^ M^−1^ s^−1^, $k_{{\mathrm{SO}}_{4}^{•-}-\mathrm{MeOH}}$) = 0.9 − 1.3 × 10^7^ M^−1^ s^−1^). As shown in Figure S2, the removal of CBZ was significantly depressed by MeOH and TBA, indicating that HO^•^ was the main radical responsible for CBZ degradation. This is contradictory to the prevailing viewpoint that SO_4_^•−^ serves as the main oxidant in the sulfite-based AOPs under acidic condition [46]. Figure S1 shows that the oxidation of HSO_3_^−^ in the presence of dissolved oxygen is a chain reaction. Wang et al. [31] found that alcohols could react with the involved radicals and interrupt the chain reaction, resulting in the decreased formation of HO^•^ and SO_4_^•−^. Thus, the quenching experiments might fail to achieve radical differentiation. Relative rate method was applied to analyze the role of HO^•^ and SO_4_^•−^ in pollutant abatement. In this method, a low concentration of probe compounds (CBZ, NB) was added, which was thought not to interrupt the chain reaction of HSO_3_^−^ oxidation. The degradation kinetics of these two compounds can be described with Equations (5) and (6).

(5) $$-\ln\frac{{\left[\mathrm{NB}\right]}_{t}}{{\left[\mathrm{NB}\right]}_{0}}{=k}_{{\mathrm{HO}}^{•}-\mathrm{NB}}\int_{0}^{t}{[\mathrm{HO}}^{•}]\mathrm{dt}$$

(6) $$-\ln\frac{{\left[\mathrm{CBZ}\right]}_{t}}{{\left[\mathrm{CBZ}\right]}_{0}}{=k}_{{\mathrm{HO}}^{•}-\mathrm{CBZ}}\int_{0}^{t}\left[{\mathrm{HO}}^{•}\right]{\mathrm{dt}+k}_{{\mathrm{SO}}_{4}^{•-}-\mathrm{CBZ}}\int_{0}^{t}\left[{\mathrm{SO}}_{4}^{•-}\right]\mathrm{dt}$$

where $\int_{0}^{t}{[\mathrm{HO}}^{•}]\mathrm{dt}$ and $\int_{0}^{t}\left[{\mathrm{SO}}_{4}^{•-}\right]\mathrm{dt}$ are defined as the time-integrated concentration of HO^•^ and SO_4_^•−^, respectively. $k_{{\mathrm{HO}}^{•}-\mathrm{NB}}$, $k_{{\mathrm{HO}}^{•}-\mathrm{CBZ}}$, and $k_{{\mathrm{SO}}_{4}^{•-}-\mathrm{CBZ}}$ are the second-order rate constants when the probe compound is reacting with the corresponding reactive species. With the experimentally determined removal of probe compounds (Figure 1 and Figure S3), along with literature-reported second-order rate constants of the radicals towards CBZ and NB (Table S1), the contribution of HO^•^ and SO_4_^•−^ to the degradation of CBZ was calculated. It should be noted that negligible NB was removed by Mn(III) alone (Figure S3), indicating that NB was recalcitrant to Mn(III). As shown in Figure 1, both HO^•^ and SO_4_^•−^ contributed to CBZ degradation, and SO_4_^•−^ played the major role [46]. Figure S4 shows that relative contribution of HO^•^ and SO_4_^•−^ to CBZ degradation are stable and independent of the dosage of HSO_3_^−^.

The increase of CBZ removal with increasing HSO_3_^−^ dosage from 0 to 250 µM should be attributed to the increased formation rate of radicals. However, besides serving as the radical precursor, HSO_3_^−^ also plays the role of radical scavenger. As shown in Figure S1, both HO^•^ and SO_4_^•−^ can be reduced by HSO_3_^−^ with the second-order rate constants of 2.7 × 10^9^ and 3.1 × 10^8^ M^−1^ s^−1^, respectively. Consequently, the removal of CBZ decreased with further increase of HSO_3_^−^ dosage from 250 to 2000 µM.

**Figure 2:**
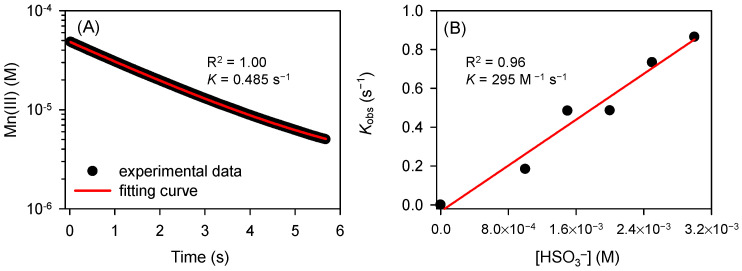
(**A**) Time course of Mn(III) (50 µM) reduction by HSO_3_^−^ (2000 µM); (**B**) linear relationship between measured pseudo-first-order rate constants (*k*_obs_, s^−1^) and HSO_3_^−^ concentrations. Conditions: [PP]_0_ = 2.5 mM, pH = 7.0.

Figure S5 shows that CBZ removal slightly increased with elevating Mn(III) dosage from 25 to 100 µM, which could be ascribed to the increased chain initiation reaction rate (i.e., the reaction of Mn(III) + HSO_3_^−^ → Mn(II) + SO_3_^•−^). Figure 2A shows a typical time course of Mn(III) reduction by HSO_3_^−^ at pH 7.0, where the initial concentrations of Mn(III) and HSO_3_^−^ were 50 and 2000 µM, respectively. The concentration of HSO_3_^−^ was assumed to be constant within 6 s under the reaction condition of this study. The loss of Mn(III) followed the pseudo-first-order kinetics, suggesting that the reaction is first-order with respect to Mn(III). For the constant initial concentration of Mn(III) (50 µM), the pseudo-first-order rate constant (*k*_obs_, s^−1^) varied linearly with HSO_3_^−^ concentration (Figure 2B), demonstrating a first-order dependence on HSO_3_^−^ concentration. Measured *k*_obs_ values for the experiments with different dosages of HSO_3_^−^ are shown in Figure S6. The reaction kinetics of HSO_3_^−^ with the PP-complexed Mn(III) can be described as

(7) $$-\frac{d\left[\mathrm{Mn}\left(\mathrm{III}\right)\right]}{\mathrm{dt}}{=k}_{\mathrm{obs}}\left[\mathrm{Mn}\left(\mathrm{III}\right)\right]{=k[\mathrm{Mn}(\mathrm{III})][\mathrm{HSO}}_{3}^{-}]$$

where *k* is the apparent second-order rate constant, which is determined to be 295 M^−1^ s^−1^ at pH 7.0. The fast reaction of Mn(III) with HSO_3_^−^ resulted in the considerable generation of radicals over a short period of time, accounting for the rapid degradation of CBZ under the reaction condition of Figure 1. Meanwhile, HSO_3_^−^ was rapidly exhausted in the presence of excessive dissolved oxygen. Though the sampling time was set to 20 s, the reaction was expected to have ended earlier.

**Figure 3:**
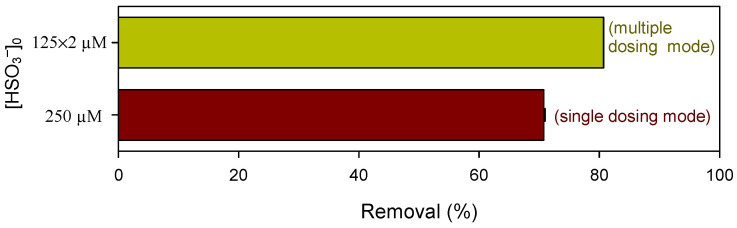
Influence of HSO_3_^−^ applied at different modes on the oxidation of CBZ by Mn(III) at pH 7.0. Conditions: [CBZ]_0_ = 5 µM, [Mn(III)]_0_ = 50 µM, [PP]_0_ = 2.5 mM. Note: multiple dosing mode represents that 125 µM of HSO_3_^−^ was dosed at the reaction time of 20 and 40 s, respectively.

To diminish the scavenging effect of HSO_3_^−^ and extend its lifetime, a multiple-dosing mode of HSO_3_^−^ was adopted. As shown in Figure 3, the degradation efficiency of CBZ achieved 81% when 125 µM of HSO_3_^−^ was added at 20 and 40 s, respectively, ~10% higher than that with a single dosing of 250 µM of HSO_3_^−^.

### 3.2. Influence of Pyrophosphate:Mn(III) Ratio of on Pollutant Abatement by Mn(III)/HSO_3_^−^ Process

**Figure 4:**
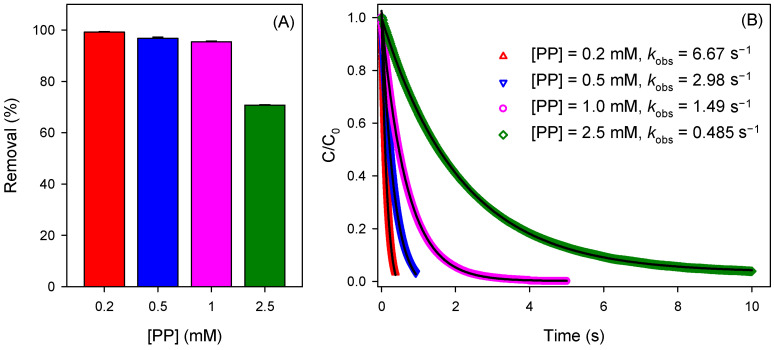
(**A**) Influence of the concentration of PP on CBZ degradation by the Mn(III)/HSO_3_^−^ process at pH 7.0; conditions: [CBZ]_0_ = 5 µM, [Mn(III)]_0_ = 50 µM, [HSO_3_^−^]_0_ = 250 µM. (**B**) The reduction kinetic of Mn(III) by HSO_3_^−^ in the presence of different concentrations of PP at pH 7.0; conditions: [Mn(III)]_0_ = 50 µM, [HSO_3_^−^]_0_ = 2 mM, the black line represents the fitting curve.

Klewicki and Morgan [47] found that PP lends a kinetic stabilization to Mn(III), which is the function of PP:Mn(III) ratios. Jiang et al. [48] disclosed that addition of PP into the MnO_4_^−^ solution enhanced the oxidation of BPA, which was attributed to the contribution of PP-stabilized Mn(III) formed in situ upon MnO_4_^−^ reduction. However, the enhancement decreased with increasing the molar ratio of Mn(III):PP from 1:10 to 1:50 at pH 6.0, indicating the decreased reactivity of Mn(III)–PP with elevating PP concentration. The influence of the concentration of PP on CBZ removal by the Mn(III)/HSO_3_^−^ process was investigated and the results are shown in Figure 4A. Though increase of PP concentration benefits the stability of Mn(III), it negatively influences CBZ removal. Figure 4B shows the reduction kinetics of PP-stabilized Mn(III) by HSO_3_^−^ as the function of PP concentration. Assuming that the concentration of HSO_3_^−^ remained constant during the reaction, the second-order rate constants of Mn(III) reacting with HSO_3_^−^ in the presence of 0.2, 0.5, 1.0, and 2.5 mM of PP were calculated to be 3335, 1490, 745, and 243 M^−1^ s^−1^, respectively, confirming the negative effect of PP on Mn(III) reactivity. Thus, a lower dosage of PP benefits the reactivity of Mn(III) and is thus in favor of CBZ degradation. Accordingly, the role of Mn(III) in natural water should be related to the species and concentrations of ligands. It should be noted that too-low concentrations of PP might lead to the disproportionation of Mn(III) spontaneously in a short time. Thus, 2.5 mM of PP was applied to prepare the stock solution of Mn(III) and conduct the following experiments.

### 3.3. Influence of Dissolved Oxygen and pH on Pollutant Degradation by Mn(III)/HSO_3_^−^ Process

**Figure 5:**
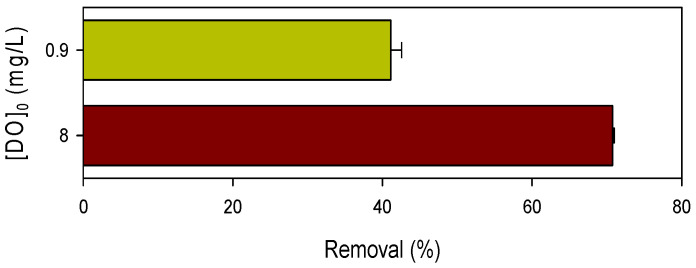
Influence of the concentration of DO on CBZ degradation by the Mn(III)/HSO_3_^−^ process. [CBZ]_0_ = 5 µM, [Mn(III)]_0_ = 50 µM, [HSO_3_^−^]_0_ = 250 µM, [PP] = 2.5 mM, pH = 7.0.

Figure S1 shows that the combination of oxygen with SO_3_^•−^ led to the formation of SO_5_^•−^ which is a critical step for the following generation of HO^•^ and SO_4_^•−^. Hence, DO is indispensable for pollutant abatement in the Mn(III)/HSO_3_^−^ process. As shown in Figure 5, decrease of DO concentration from 8.0 to 0.9 mg/L depressed the removal efficiency of CBZ by 42%. According to the stoichiometric ratio of 1:1 in the reaction O_2_ with SO_3_^•−^, ~28 µM of SO_5_^•−^ was expected to be formed in the presence of 0.9 mg/L of DO, much lower than that in the presence of 8.0 mg/L of DO. Though the transformation efficiency from SO_5_^•−^ to HO^•^ and SO_4_^•−^ is not clear, the limited formation of SO_5_^•−^ is considered to be the main factor for the low removal efficiency of pollutant in the presence of low concentration of DO. The influence of DO on the pollutant degradation further demonstrated that Mn(III) chiefly played the role of triggering oxidation of HSO_3_^−^ by oxygen which involved the generation of highly reactive species.

**Figure 6:**
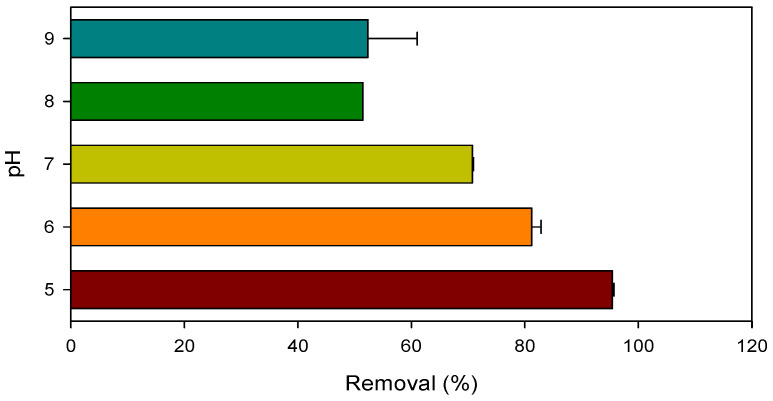
Removal of CBZ by the Mn(III)/HSO_3_^−^ process under the pH range of 5.0–9.0. Conditions: [CBZ]_0_ = 5 µM, [NB]_0_ = 1 µM, [Mn(III)]_0_ = 50 µM, [PP]_0_ = 2.5 mM, [HSO_3_^−^]_0_ = 250 µM.

The influence of pH on the degradation of CBZ in the Mn(III)/HSO_3_^−^ process was investigated, and the results are shown in Figure 6. Over 95% of CBZ was removed at pH 5.0, while the removal of ciprofloxacin dropped progressively from 81% to 52% as pH increased from 6.0 to 9.0. The reduction kinetics of Mn(III) by HSO_3_^−^ under the pH range of 5.0–9.0 were displayed in Figure S7. The second-order rate constants of Mn(III) reacting with HSO_3_^−^ at pH 5.0–9.0 were calculated and are listed in Table S2. The reaction rate of Mn(III) with HSO_3_^−^ is sensitive to pH and decreased monotonously with increasing pH. The p*K*_a_ of HSO_3_^−^ is 7.2, and the species of HSO_3_^−^ shifted to SO_3_^2−^ with increasing pH from 5.0 to 9.0. The electron density on the SO_3_^2−^ is much higher than that on the HSO_3_^−^ and the oxidation of SO_3_^2−^ is expected to be easier than that of HSO_3_^−^. Thus, the declined reaction rate of Mn(III) with HSO_3_^−^ with increasing pH should be mainly ascribed to the decreased reactivity of Mn(III), which is consistent with previous studies on the relationship of Mn(III) reactivity with pH [28,49,50]. Consequently, the degradation efficiency of CBZ is prominent at low pH due to the higher formation rate of radicals. Table S3 summarizes the treatment efficiency of CBZ by different HSO_3_^−^-based AOPs. Compared to other reported HSO_3_^−^-based AOPs (Table S3), the Mn(III)/HSO_3_^−^ process is more superior in pollutant removal.

### 3.4. Influence of Chloride and DOM on Pollutant Degradation by Mn(III)/HSO_3_^−^

**Figure 7:**
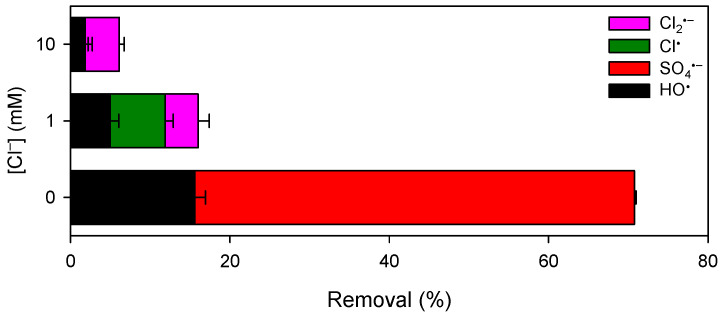
Influence of Cl^−^ on CBZ degradation by the Mn(III)/HSO_3_^−^ process. [CBZ]_0_ = 5 µM, [Mn(III)]_0_ = 50 µM, [HSO_3_^−^]_0_ = 250 µM, [PP] = 2.5 mM, pH = 7.0.

Previous studies have demonstrated that Cl^−^, one of the most common background water constituents, shifts the distribution of SO_4_^•−^-based AOPs due to the high reactivity of Cl^−^ towards SO_4_^•−^ [51,52]. Consequently, the treatment efficiency of SO_4_^•−^-based AOPs decreased with increasing the dosage of Cl^−^. Figure 7 shows the removal of CBZ by the Mn(III)/HSO_3_^−^ process in the presence of different concentrations of Cl^−^. Consistent with previous studies [51,52,53], addition of 1 and 10 mM of Cl^−^ retarded the degradation of CBZ, and the negative effect was more obvious with higher dosage of Cl^−^. Table S4 summarizes the principal reactions in the Cl^−^/SO_4_^•−^ system along with their rate constants obtained from the literature. The conversion of radicals by Cl^−^ complicates this process and results in the generation of multiple secondary radicals (e.g., HO^•^, Cl^•^, Cl_2_^•−^, and ClO^•^). It should be noted that ClO^•^ was derived from the radicals (e.g., HO^•^, Cl^•^, and Cl_2_^•−^) with HOCl. The accumulation of HOCl was found to be negligible, which might be ascribed to the rapid consumption of HOCl by the added HSO_3_^−^ (Figure S8). Thus, ClO^•^ was neglected in this study. In addition, due to the fast consumption of SO_4_^•−^ by Cl^−^, the concentration of SO_4_^•−^ decreased significantly in the presence of Cl^−^ at the level of mM and its contribution to pollutant abatement was commonly ignored [51]. Therefore, HO^•^, Cl^•^, and Cl_2_^•−^ were considered to be the major reactive species responsible for pollutant degradation.

CBZ, NB, and BPA were employed to analyze the distribution of these radicals. BPA was recalcitrant to Mn(III) (Figure S9). The degradation kinetics of the probe compound by the Mn(III)/HSO_3_^−^ process in the presence of Cl^−^ can be descried by Equations (5), (8) and (9).

(8) $$-\ln\frac{{\left[\mathrm{CBZ}\right]}_{t}}{{\left[\mathrm{CBZ}\right]}_{0}}{=k}_{{\mathrm{HO}}^{•}-\mathrm{CBZ}}\int_{0}^{t}\left[{\mathrm{HO}}^{•}\right]{\mathrm{dt}+k}_{{\mathrm{Cl}}^{•}-\mathrm{CBZ}}\int_{0}^{t}\left[{\mathrm{Cl}}^{•}\right]{\mathrm{dt}+k}_{{\mathrm{Cl}}_{2}^{•-}-\mathrm{CBZ}}\int_{0}^{t}\left[{\mathrm{Cl}}_{2}^{•-}\right]\mathrm{dt}$$

(9) $$-\ln\frac{{\left[\mathrm{BPA}\right]}_{t}}{{\left[\mathrm{BPA}\right]}_{0}}{=k}_{{\mathrm{HO}}^{•}-\mathrm{BPA}}\int_{0}^{t}\left[{\mathrm{HO}}^{•}\right]{\mathrm{dt}+k}_{{\mathrm{Cl}}^{•}-\mathrm{BPA}}\int_{0}^{t}\left[{\mathrm{Cl}}^{•}\right]{\mathrm{dt}+k}_{{\mathrm{Cl}}_{2}^{•-}-\mathrm{BPA}}\int_{0}^{t}\left[{\mathrm{Cl}}_{2}^{•-}\right]\mathrm{dt}$$

where $k_{{\mathrm{Cl}}^{•}-\mathrm{CBZ}}$ and $k_{{\mathrm{Cl}}_{2}^{•-}-\mathrm{CBZ}}$ are the second-order rate constants of CBZ oxidation by Cl^•^ and Cl_2_^•−^, respectively. $k_{{\mathrm{HO}}^{•}-\mathrm{BPA}}$, $k_{{\mathrm{Cl}}^{•}-\mathrm{BPA}}$, and $k_{{\mathrm{Cl}}_{2}^{•-}-\mathrm{BPA}}$ are the second-order rate constants of BPA oxidation by HO^•^, Cl^•^, and Cl_2_^•−^, respectively. $\int_{0}^{t}[{\mathrm{Cl}}^{•}]\mathrm{dt}$ and $\int_{0}^{t}\left[{\mathrm{Cl}}_{2}^{•-}\right]\mathrm{dt}$ are the time-integrated concentration of Cl^•^ and Cl_2_^•−^, respectively. Based on the removal of these probe compounds (Figure 7 and Figure S10) and the listed second-order rate constants in Table S1, the time-integrated concentrations of HO^•^, Cl^•^, and Cl_2_^•−^ were calculated and are shown in Figure S11. The concentration of HO^•^ decreased with increasing Cl^−^ dosage from 0 to 10 mM. It is traditionally thought that the Cl^•^ formed from the reaction of Cl^−^ with SO_4_^•−^ tends to combine with H_2_O/HO^−^ to produce ClOH^•−^ which subsequent decomposes to HO^•^ and Cl^−^, resulting in the increase of HO^•^ concentration. Thus, the steady-state concentration of HO^•^ was commonly observed to increase by several times in the persulfate-based AOPs after dosing 1‒10 mM of Cl^−^ [51]. Different to persulfate, HSO_3_^−^ also plays the role of radical scavenger and possesses high reactivity towards HO^•^ (*k* = 2.7 × 10^9^ M^−1^ s^−1^) [45], which might be the reason for the decreased concentration of HO^•^ with dosing Cl^−^ in the Mn(III)/HSO_3_^−^ process. In addition, though the reactivity of ClOH^•−^ towards HSO_3_^−^ was unknown, this reaction pathway of ClOH^•−^ might also depress the formation of HO^•^. The value of $\int_{0}^{t}[{\mathrm{Cl}}^{•}]\mathrm{dt}$ achieved 2.3 × 10^−12^ M·s in the presence of 1 mM of Cl^−^ while decreased to 0 in the presence of 10 mM of Cl^−^ (Figure S11). This could be ascribed to the high reactivity of Cl^•^ towards Cl^−^ (eq s2 in Table S4), leading to the conversion of Cl^•^ to Cl_2_^•−^ in the presence of high concentration of Cl^−^. The increased concentration of Cl_2_^•−^ with increasing dosage of Cl^−^ further confirmed this speculation. The time-integrated concentration of Cl_2_^•−^ is much higher than that of other radicals, resulting in the predominant contribution of Cl_2_^•−^ in CBZ abatement (Figure S11 and Figure 7). Besides oxidizing CBZ, Cl_2_^•−^ is also expected to transform HSO_3_^−^ to SO_3_^•−^, resulting in the enhanced consumption rate of HSO_3_^−^. Consequently, the removal efficiency of CBZ decreased.

Chlorate is a typical byproduct of the persulfate-based AOPs in the presence of chloride [54]. However, negligible chlorate was detected in the Mn(III)/HSO_3_^−^ process in the presence of 1 and 10 mM of chloride (Figure S8), suggesting that chlorate formation is not a concern in the Mn(III)/HSO_3_^−^ process.

**Figure 8:**
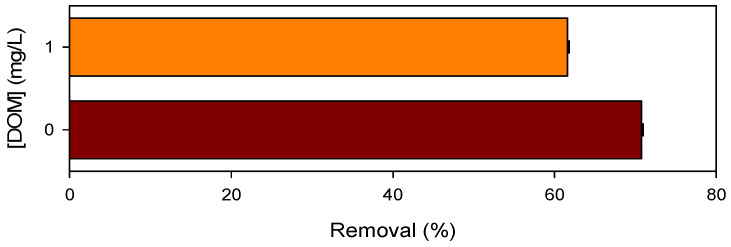
Influence of DOM on CBZ degradation by the Mn(III)/HSO_3_^−^ process. [CBZ]_0_ = 5 µM, [Mn(III)]_0_ = 50 µM, [HSO_3_^−^]_0_ = 250 µM, [PP] = 2.5 mM, pH = 7.0.

DOM is one of the most common background water constituents in natural water and often shows negative effects on pollutant abatement by AOPs [55]. The influence of DOM on CBZ removal by the Mn(III)/HSO_3_^−^ process was shown in Figure 8. A total of 1.0 mg/L of DOM, the representative concentration in natural water, decreased CBZ removal by 13%. DOM could serve as the radical scavenger (Table S5), and resulted in the decrease of CBZ removal. In addition, the conversion of radicals by DOM might interrupt the chain propagation of HSO_3_^−^ oxidation, and thus decrease the consumption of HSO_3_^−^. As shown in Figure S12, DOM retarded the oxidation of HSO_3_^−^. As mentioned above, that HSO_3_^−^ also plays the role of radical scavenger, the retarded consumption of HSO_3_^−^ in the presence of DOM might take some responsibility in decreasing CBZ removal by the Mn(III)/HSO_3_^−^ process under the reaction condition of this study.

## 4. Conclusions

PP-complexed Mn(III) was proven to be effective in activating HSO_3_^−^ in this study. Due to the high reactivity of Mn(III) towards HSO_3_^−^, considerable concentrations of SO_4_^•−^ and HO^•^ were generated rapidly, resulting in the effective removal of pollutants. The dosage of HSO_3_^−^ was critical for pollutant abatement due to the scavenging effect of HSO_3_^−^. Increasing pH and PP:Mn(III) ratio depressed the reactivity of Mn(III) towards HSO_3_^−^ and thus inhibited pollutant removal. Similar to other SO_4_^•−^-based advanced oxidation processes, a significant loss of the treatment efficiency induced by Cl^−^ was observed. In addition, Cl^−^ complicated the chemistry of the Mn(III)/HSO_3_^−^ system and introduced reactive chlorine species. Another common water constituent, DOM, also showed negative effect on the treatment efficiency of the Mn(III)/HSO_3_^−^ process. This study broadened the HSO_3_^−^-based advanced oxidation processes. However, water parameters and constituents need careful consideration in the application of the Mn(III)/HSO_3_^−^ process for pollutant abatement in real water. Different ligands might exist in water and influence the reactivity of Mn(III) towards HSO_3_^−^, which also needs attention in conducting the Mn(III)/HSO_3_^−^ process.

## Data Availability

The data presented in this study are available on request from the corresponding author.

## Supplementary Materials

The following supporting information can be downloaded at: https://www.mdpi.com/article/10.3390/ijerph19159437/s1. Figure S1: The reported mechanism for the evolution of radicals in the transition metal/HSO_3_^–^ process; Figure S2: Influence of radical scavengers on CBZ removal; Figure S3: Influence of HSO_3_^–^ dosage on NB degradation; Figure S4: The relationship of the contribution of SO_4_^•–^ and HO^•^ on CBZ degradation; Figure S5: Influence of Mn(III) dosage on CBZ removal; Figures S6 and S7: The reduction kinetic of Mn(III) at different conditions; Figure S8: The spectra of ion chromatography of reaction solution; Figure S9: Degradation of BPA by Mn(III) alone; Figure S10: Influence of Cl^–^ on the degradation of BPA and NB by Mn(III)/HSO_3_^–^ process; Figure S11: Influence of Cl^–^ on the distribution of radicals; Figure S12: Influence of DOM on the consumption of HSO_3_^–^ in the presence of Mn(III); Table S1: Second-order rate constants of pollutant oxidation by radicals; Table S2: The rate constants of Mn(III) reacting with HSO_3_^–^; Table S3: Removal of CBZ by different HSO_3_^–^-based AOPs; Table S4: Principal reactions in the Cl^–^/SO_4_^•–^ system; Table S5: Second-order rate constants of DOM oxidation by different radicals. References [29,45,46,51,55,56,57,58,59,60,61,62] are cited in the supplementary materials
