# Supplementary material for: Activation of Bisulfite with Pyrophosphate-Complexed Mn(III) for Fast Oxidation of Organic Pollutants
Source: Int J Environ Res Public Health. 2022 Aug 1;19(15):9437. doi: 10.3390/ijerph19159437 (PMC9368537; doi:10.3390/ijerph19159437)
Supplement: Supplementary file 1 [file ijerph-19-09437-s001.zip › ijerph-1817028-supplementary.pdf]

SUPPLEMENTARY DATA

Activation of bisulfite with pyrophosphate-complexed Mn(III) for fast oxidation of organic pollutants

Qianli Guo[1], Xianhu Qi[1], Jian Zhang[1,2], Bo Sun[1,*]

[1] Shandong Key Laboratory of Water Pollution Control and Resource Reuse, School of Environmental Science & Engineering, Shandong University, Qingdao 266237, China

[2] College of Safety and Environmental Engineering, Shandong University of Science and Technology, Qingdao 266590, China

* Corresponding author:

Dr. Bo Sun. E-mail: sdusunbo@sdu.edu.cn

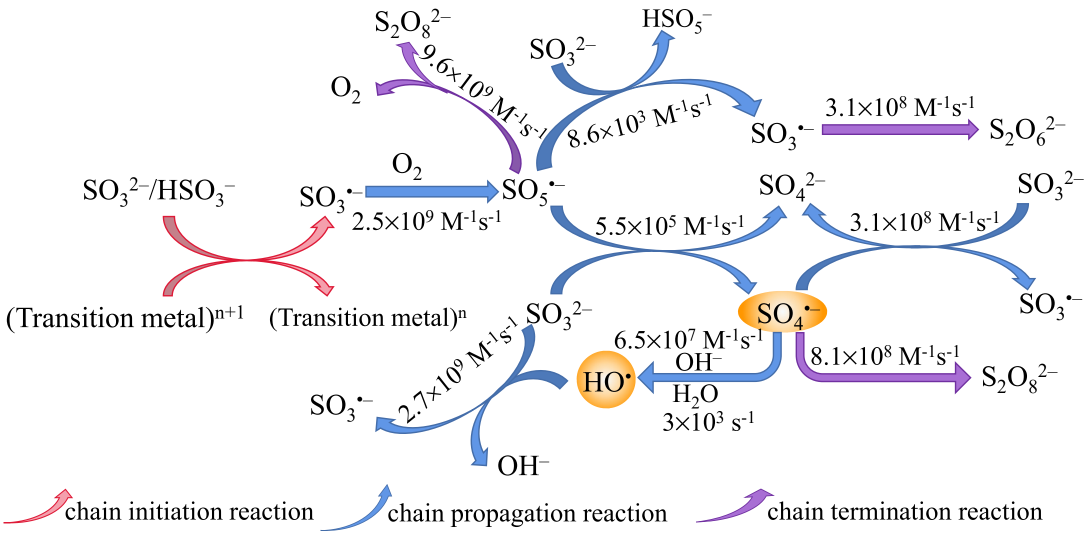

Figure S1 The reported mechanism for the evolution of radicals in the transition metal/HSO$_3^-$ process [45].

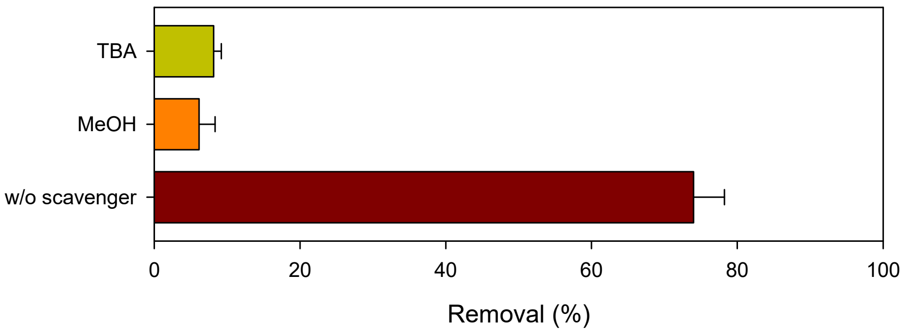

Figure S2 Influence of MeOH and TBA on the removal of CBZ by the Mn(III)/HSO$_3^-$ process at pH 7.0. Conditions: [CBZ]$_0$ = 5 μM, [Mn(III)]$_0$ = 50 μM, [PP]$_0$ = 2.5 mM, [HSO$_3^-$]$_0$ = 250 μM, [MeOH] = 0.1 M, [TBA] = 0.1 M.

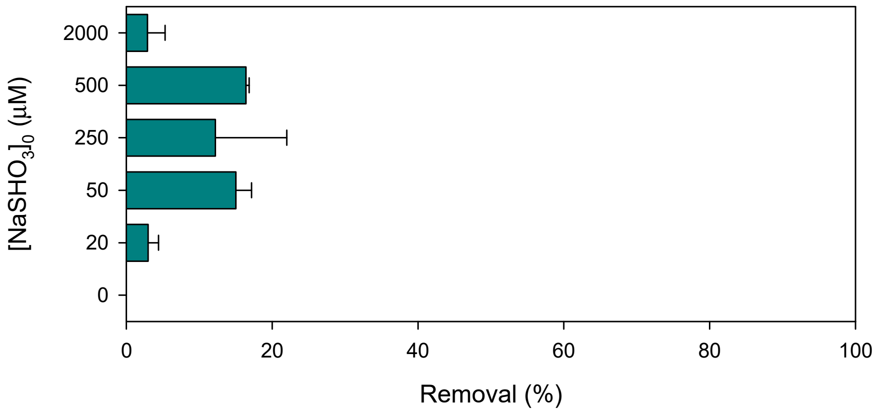

Figure S3 Degradation of NB by Mn(III) in the presence of different concentrations of HSO$_3^-$. Conditions: [CBZ]$_0$ = 5 μM, [NB]$_0$ = 1 μM, [Mn(III)]$_0$ = 50 μM, [PP]$_0$ = 2.5 mM, pH = 7.0.

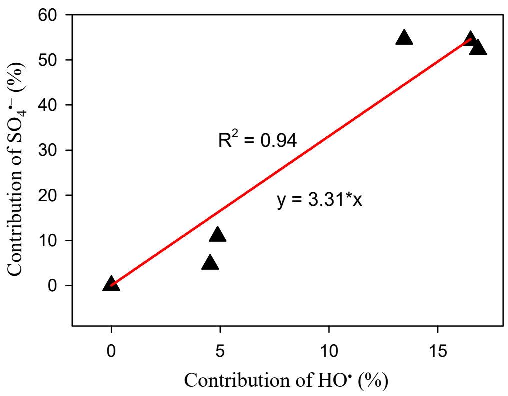

Figure S4 The relationship of the contribution of $SO_4^{\bullet -}$ and $HO^{\bullet}$ on CBZ degradation. Conditions: $[CBZ]_0$ = 5 μM, $[NB]_0$ = 1μM, $[Mn(III)]_0$ = 50 μM, $[PP]_0$ = 2.5 mM, pH = 7.0.

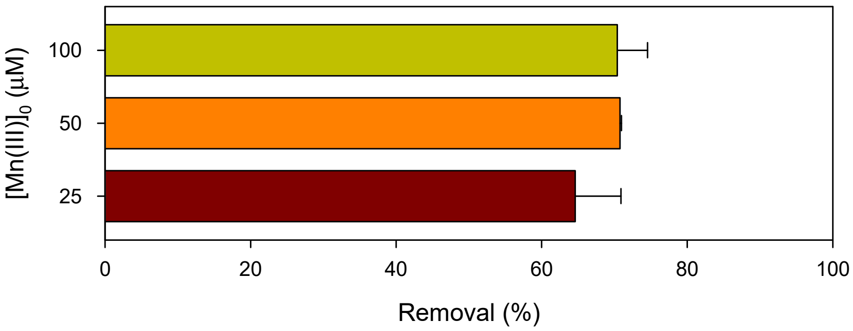

Figure S5 Influence of the dosage of Mn(III) on the removal of CBZ by Mn(III)/HSO_3^- process at pH 7.0. Conditions: $[CBZ]_0$ = 5 μM, $[NB]_0$ = 1 μM, $[HSO_3^-]_0$ = 250 μM, $[PP]_0$ = 2.5 mM.

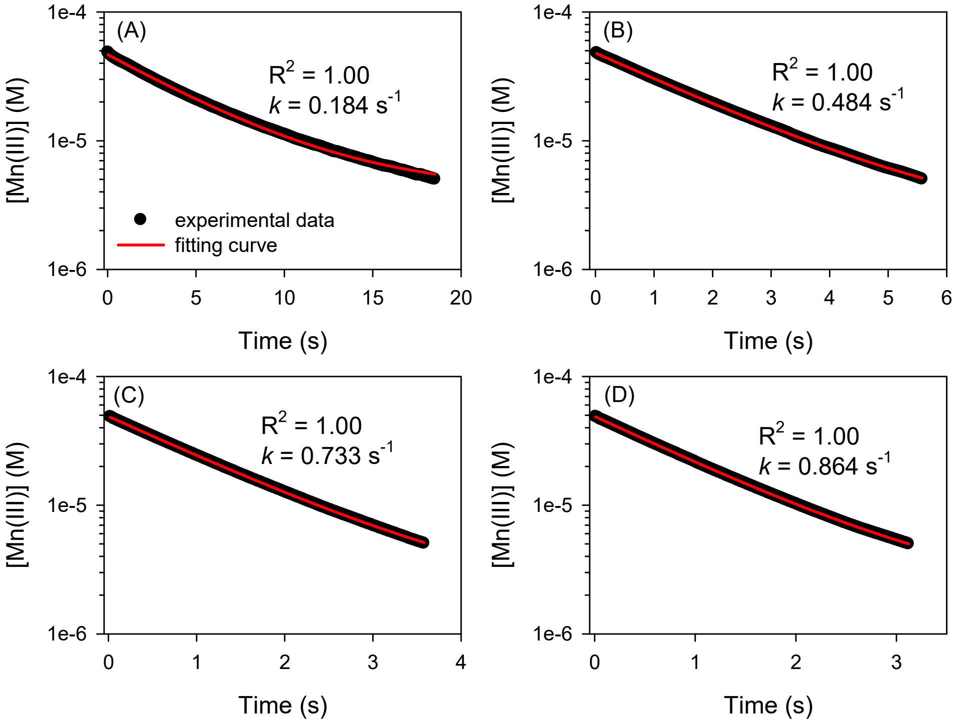

Figure S6 Time course of Mn(III) reduction by 1 mM (A), 1.5 mM (B), 2.5 mM (C) and 3 mM (D) of HSO_3^- pH7.0. Conditions: $[Mn(III)]_0$ = 50 μM, $[PP]_0$ = 2.5 mM.

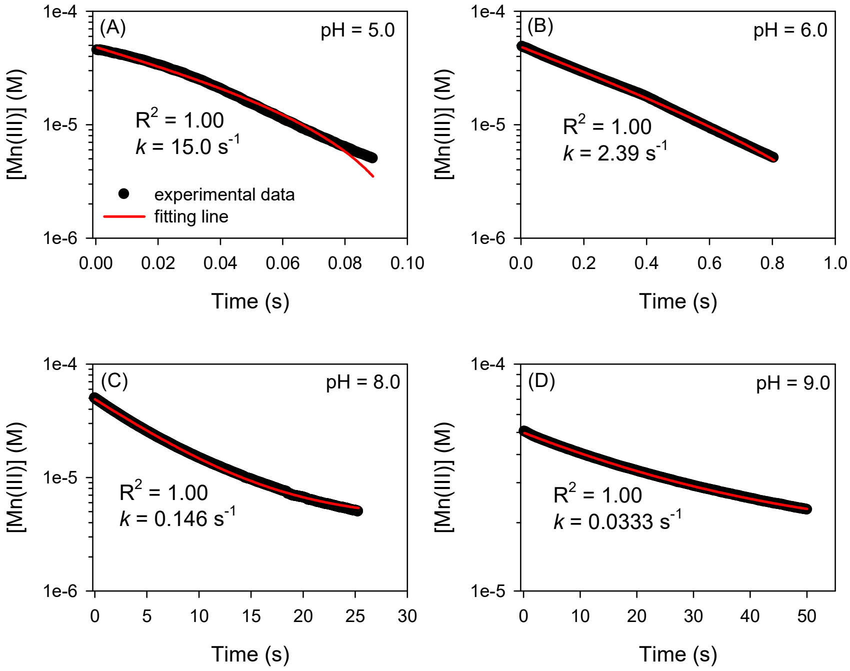

Figure S7 Time course of Mn(III) reduction by HSO_3^- at different pH. Conditions: $[Mn(III)]_0$ = 50 μM, $[HSO_3^-]_0$ = 2 mM, $[PP]_0$ = 2.5 mM.

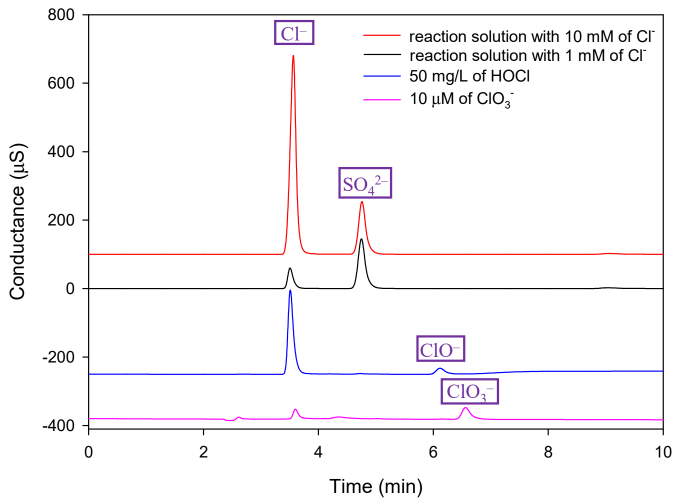

Figure S8 The spectra of ion chromatography of different solutions. Note: reaction solution represents the solution after reaction of 50 μM of Mn(III) with 250 μM of HSO_3^- at pH 7.0.

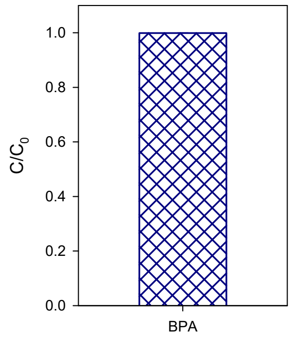

Figure S9 Degradation of BPA by Mn(III) alone. Conditions: $[BPA]_0 = 5$ μM, $[Mn(III)]_0 = 50$ μM, $[PP]_0 = 2.5$ mM, pH = 7.0.

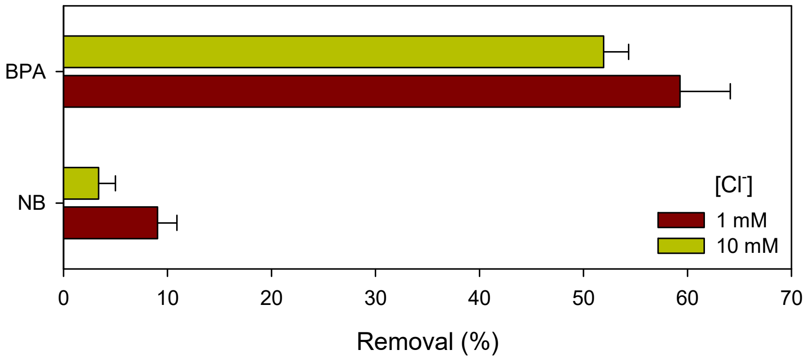

Figure S10 Degradation of BPA and NB by Mn(III)/HSO_3^- process in the presence of different concentrations of Cl^-. Conditions: $[CBZ]_0 = 5$ μM, $[NB]_0 = 1$ μM, $[HSO_3^-]_0 = 250$ μM, $[PP]_0 = 2.5$ mM, pH = 7.0.

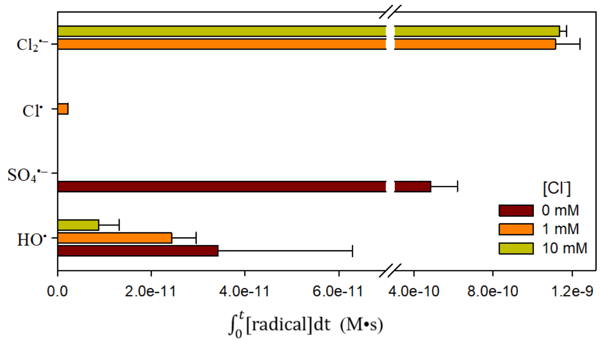

Figure S11 Influence of Cl^- on the accumulated concentrations of radicals in Mn(III)/HSO_3^- process. Conditions: $[CBZ]_0 = 5$ μM, $[NB]_0 = 1$ μM, $[BPA]_0 = 5$ μM, $[HSO_3^-]_0 = 250$ μM, $[PP]_0 = 2.5$ mM, pH = 7.0.

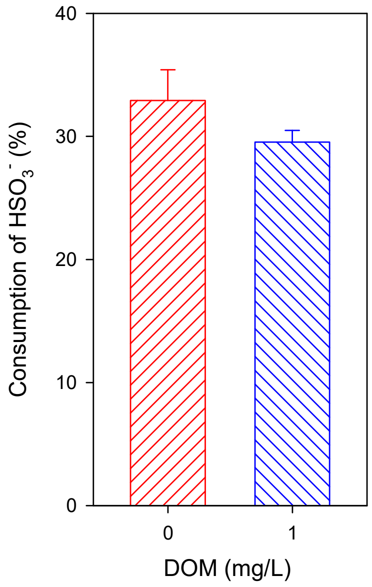

Figure S12 Influence of DOM on the consumption of HSO_3^- in the presence of Mn(III). Conditions: $[CBZ]_0 = 5$ μM, $[HSO_3^-]_0 = 2$ mM, $[Mn(III)]_0 = 50$ μM, $[PP]_0 = 2.5$ mM, pH = 7.0.

Table S1 Second-order rate constants of the $HO^{\bullet}$ and RCS.

	$k_{HO^{\bullet}}$ (10^9 $M^{-1}s^{-1}$)	$k_{Cl^{\bullet}}$ (10^9 $M^{-1}s^{-1}$)	$k_{Cl_2^{\bullet-}}$ (10^9 $M^{-1}s^{-1}$)
BPA	8.77	18.2	0.58
NB	3.9	Negligible	Negligible
CBZ	8.8	33.0	0.043

Table S2 Second-order rate constants of Mn(III) reacting with HSO_3^- at different pH.

pH	k (M^{-1} s^{-1})
5.0	7500
6.0	1195
7.0	295
8.0	73
9.0	17

Conditions: $[Mn(III)]_0$ = 50 μM, $[HSO_3^-]_0$ = 2 mM, $[PP]_0$ = 2.5 mM.

Table S3 Removal of carbamazepine by different HSO_3^--based AOPs.

Carbamazepine	System	Conditions	Reaction time	Reference
95%	$Mn(III)/HSO_3^-$	$[Mn(III)]_0$ = 50 μM, $[HSO_3^-]_0$ = 250 μM, $[CBZ]_0$ = 5μM, pH_{ini} = 5.0.	20 s	this study
60%	MnO_2/HSO_3^-	$[MnO_2]_0$ = 50 μM, $[HSO_3^-]_0$ = 750 μM, $[CBZ]_0$ = 5μM, pH_{ini} = 5.0.	10 min	[29]
67%	MnO_4^-/HSO_3^-	$[MnO_4^-]_0$ = 50 μM, $[HSO_3^-]_0$ = 250μM, $[CBZ]_0$ = 5μM, pH_{ini} = 3.0.	not given	[45]
66%	$Fe(III)/HSO_3^-$	$[Fe(III)]_0$ = 100 μM, $[HSO_3^-]_0$ = 500μM, $[CBZ]_0$ = 5μM, pH_{ini} = 3.0.	200 s	[46]

Table S4 Principal reactions in the $Cl^-/SO_4^{\bullet-}$ system.

NO.	chemical reaction	rate constants	reference
s1	$Cl^- + SO_4^{\bullet-} \rightarrow SO_4^{2-} + Cl^\bullet$	3.0×10^8 M^{-1} s^{-1}	[56]
s2	$Cl^\bullet + Cl^- \rightarrow Cl_2^{\bullet-}$	8.50×10^9 M^{-1} s^{-1}	[57]
s3	$Cl^\bullet + Cl^\bullet \rightarrow Cl_2$	8.80×10^7 M^{-1} s^{-1}	[57]
s4	$Cl_2^{\bullet-} \rightarrow Cl^\bullet + Cl^-$	6.00×10^4 s^{-1}	[57]
s5	$2Cl_2^{\bullet-} \rightarrow Cl_2 + 2Cl^-$	9.0×10^8 M^{-1} s^{-1}	[57]
s6	$Cl_2^{\bullet-} + Cl^\bullet \rightarrow Cl_2 + Cl^-$	2.10×10^9 M^{-1} s^{-1}	[57]
s7	$Cl_2 + H_2O \rightarrow Cl^- + HOCl + H^+$	15 M^{-1} s^{-1}	[58]
s8	$Cl^- + HOCl + H^+ \rightarrow Cl_2 + H_2O$	1.8×10^{-2} M^{-2} s^{-1}	[58]
s9	$HOCl + Cl^\bullet \rightarrow ClO^\bullet + H^+ + Cl^-$	3.0×10^9 M^{-1} s^{-1}	[59]
s10	$HOCl + HO^\bullet \rightarrow ClO^\bullet + H_2O$	2.0×10^9 M^{-1} s^{-1}	[60]
s11	$OCl^- + Cl_2^{\bullet-} \rightarrow ClO^\bullet + 2Cl^-$	2.9×10^8 M^{-1} s^{-1}	[61]
s12	$2ClO^\bullet + H_2O \rightarrow HOCl + H^+ + ClO_2^-$	2.5×10^9 M^{-1} s^{-1}	[59]

Table S5 Second-order rate constants of the DOM oxidation by different radicals

radical	*k*	reference
$HO^\bullet$	2.56×10^6 $(g_C\ L^{-1})^{-1}$ s^{-1}	[55]
$\mathbf{SO_4^{\bullet-}}$	6.80×10^5 $(g_C\ L^{-1})^{-1}$ s^{-1}	[55]
$Cl^\bullet$	1.3×10^7 $(g_C\ L^{-1})^{-1}$ s^{-1}	[62]
$\mathbf{Cl_2^{\bullet-}}$	1.1×10^6 $(g_C\ L^{-1})^{-1}$ s^{-1}	[51]
